# Healthy lifestyle gone bad: effect of the COVID-19 pandemic on the daily habits of children and adolescents with type 1 diabetes

**DOI:** 10.20945/2359-3997000000490

**Published:** 2022-06-02

**Authors:** Giovana B. de Oliveira, Janine Alessi, Isadora Nunes Erthal, Julia Belato Teixeira, Milena Sbalchiero Morello, Raquel Jaqueline Eder Ribeiro, Guilherme H. Telo, Beatriz D. Schaan, Gabriela H. Telo

**Affiliations:** 1 Pontifícia Universidade Católica do Rio Grande do Sul Faculdade de Medicina Porto Alegre RS Brasil Faculdade de Medicina, Pontifícia Universidade Católica do Rio Grande do Sul, Porto Alegre, RS, Brasil; 2 Universidade Federal do Rio Grande do Sul Programa de Ciências Médicas: Endocrinologia Porto Alegre RS Brasil Programa de Ciências Médicas: Endocrinologia, Universidade Federal do Rio Grande do Sul, Porto Alegre, RS, Brasil; 3 Pontifícia Universidade Católica do Rio Grande do Sul Hospital São Lucas Departamento de Medicina Interna Porto Alegre RS Brasil Departamento de Medicina Interna, Hospital São Lucas, Pontifícia Universidade Católica do Rio Grande do Sul, Porto Alegre, RS, Brasil; 4 Pontifícia Universidade Católica do Rio Grande do Porto Alegre Programa de Medicina e Ciências da Saúde Porto Alegre RS Brasil Programa de Medicina e Ciências da Saúde, Pontifícia Universidade Católica do Rio Grande do Porto Alegre, RS, Brasil; 5 Hospital de Clínicas de Porto Alegre Divisão de Cardiologia Porto Alegre RS Brasil Divisão de Cardiologia, Hospital de Clínicas de Porto Alegre, Porto Alegre, Porto Alegre, RS, Brasil; 6 Universidade Federal do Rio Grande do Sul Faculdade de Medicina Porto Alegre RS Brasil Faculdade de Medicina, Universidade Federal do Rio Grande do Sul, Porto Alegre, RS, Brasil; 7 Hospital de Clínicas de Porto Alegre Divisão de Endocrinologia Porto Alegre RS Brasil Divisão de Endocrinologia, Hospital de Clínicas de Porto Alegre, Porto Alegre, RS, Brasil

**Keywords:** Eating habits, screen time, physical activity, treatment adherence, behavior

## Abstract

**Objective:**

To assess caregivers’ perception about the changes in the daily habits of children and adolescents with type 1 diabetes during the COVID-19 pandemic.

**Subjects and methods:**

Primary caregivers of youth aged ≤18 with or without type 1 diabetes were selected for the diabetes and the control groups. Caregivers estimated the youth's time (hours) of physical activity and screen time before and during the pandemic, and rated the quality of eating habits and medication adherence from 0 to 10. The primary outcome was the change in physical activity time, screen time, and eating habits scores during isolation. Between-group analyses and within-group comparisons were conducted. A post hoc analysis was performed using logistic regression to correct for confounding factors.

**Results:**

In total, 764 participants were included (381 diabetes group vs. 383 control group). Before the pandemic, the diabetes group presented a reduced median of physical activity (P < 0.001) and screen time (P < 0.001). During the pandemic, the difference between both groups remained similar (P = 0.58). Scores of quality of eating habits were similar in both groups before the pandemic [8.0 (7.0-9.0) vs. 8.0 (7.0-9.0), P = 0.31] but decreased during the pandemic [7.0 (5.1-8.1) vs. 8.0 (6.0-9.0), P < 0.001]. The diabetes group had a significantly worse change in eating habits scores (P < 0.01).

**Conclusion:**

During the pandemic, eating habits were significantly worse in youth with diabetes than in those without diabetes.

## INTRODUCTION

Over 1.1 million children and adolescents younger than 20 years live with type 1 diabetes mellitus (1), an autoimmune disease with a worldwide prevalence of 9.5% (2) and incidence rate that has been increasing by approximately 2%-5% (3,4). Youth with type 1 diabetes must adhere to treatment based on a healthy and well-established lifestyle for an adequate glycemic control. Overall, physical activity is associated with improved disease status, although glycemic response to exercise may vary in each individual with type 1 diabetes (5,6). The American Diabetes Association recommends at least 60 minutes a day of moderate aerobic exercise and weight training three times a week (6). Also, following a balanced diet and limiting carbohydrates and fats consumption is essential to avoid both hyper and hypoglycemia. Besides facing the challenges regarding the upbringing and education of children and adolescents, families responsible for youth with type 1 diabetes should also help control the disease and maintain the child's healthy lifestyle (7).

In Brazil, the COVID-19 pandemic started in March 2020, significantly changing family dynamics. These changes were noticed worldwide, affecting parents or caregivers of children and adolescents in most countries affected by the new coronavirus. In a literature review, Prime and cols. show that the pandemic has affected the welfare of populations worldwide, being more than a crisis of public health and economic stability: it has negatively impacted on the well-being of many families, and caregivers are pressured to maintain a stable household despite all adversities (8). Social distancing and home isolation imply changes in the daily routine of families responsible for children and adolescents, possibly causing great psychological and emotional burden (9), especially in this younger group (10). For families of youth with type 1 diabetes, who must maintain a well-established routine for adequate glycemic control, the changes brought by the pandemic cause even greater stress. School closure as well as the closing of parks and recreation rooms interferes in the social life of the youth and completely changes the routine of children or adolescents with diabetes, modifying their eating habits and reducing their daily physical activity (11). The longitudinal study of Pietrobelli and cols. show that diet, physical activity, and sleeping habits of children and adolescents with obesity were negatively affected after three weeks of confinement during the lockdown period (12). Important changes in diet, physical exercise, and daily habits overall can significantly impact the glycemic control of youth with type 1 diabetes, making their blood glucose unpredictable and impairing their previously standardized and well-established routine of insulin use.

Despite its importance, few studies have prioritized assessing the routine of healthy habits in crises such as the current COVID-19 pandemic. A recently published study on the changes of food choice in Polish adolescents showed that, during the pandemic, this population may have changed the food choice determinants and increased the importance of health and weight control, reflecting a positive change in youth without diabetes (13). Another study assessed how the pandemic modified dietary trends of youth from Spain, Italy, Brazil, Colombia, and Chile, also showing a positive change in eating habits, with increased legume, fruit, and vegetable intake (14). These results were associated with increased time to cook, which can improve eating habits. However, the authors found that this change did not improve the diet quality overall, since the increased healthy food intake was followed by a higher consumption of sweets, likely from boredom and stress (14). To date, no studies have assessed if the pandemic changes in lifestyle are more significant in children or adolescents with type 1 diabetes than in those without the disease. This study aimed to assess the perception of caregivers about the changes in the daily habits of children and adolescents with type 1 diabetes during the COVID-19 pandemic.

## SUBJECTS AND METHODS

### Study design and setting

A cross-sectional web-based study was performed to assess the perceptions of caregivers about the change of habits in children and adolescents with type 1 diabetes during the COVID-19 pandemic. Parents and primary caregivers of youth with type 1 diabetes (diabetes group) and without diabetes (control group) were invited to participate in the study. The diabetes group was enrolled via social media of the Brazilian *Associação de Diabetes Juvenil*. The control group was enrolled via social media of student university leagues in Brazil. An electronic invitation was sent for participants two months after social distancing measures began in Brazil. For the proposed evaluation, an online questionnaire was applied addressing the aspects of interest. The research team had no personal contact with participants to protect them from unnecessary exposure during the COVID-19 pandemic. The informed consent forms were electronically signed by individuals aged over 18 years before initiating the questionnaire. The research team had no direct contact with minors, only their guardians or caregivers. The project was approved by the Research Ethics Committee of the main researcher's institution (number 4.045.411, CAAE 31160420.2.0000.5336). The manuscript description follows the STROBE guideline (13).

### Participants

Adults of any age who were primary caregivers of youth aged ≤18 years with type 1 diabetes were selected for the diabetes group. For the control group, adults who were primary caregivers of youth without diabetes were selected. Exclusion criteria included filling out less than 75% of the online questionnaire.

### Variables and data source

An online questionnaire was developed to assess the perception of caregivers about changes in habits during the COVID-19 pandemic. To better understand the data obtained, participants were approached in two steps:

#### First step:

A detailed assessment of the participants’ sociodemographic characteristics was conducted, including age, ethnicity, sex, family income, and region of origin. In the diabetes group, the youth under care was assessed on age, diabetes duration, and presence of chronic complications. Caregivers in the control group (non-diabetes) were asked if the children had chronic diseases. Finally, aspects related to the COVID-19 pandemic were assessed, including the compliance with social distancing measures, suspension of school activities, and possible financial or medical care difficulties due to the pandemic.

#### Second step:

To assess changes in habits, the caregivers’ perception of children and adolescents were evaluated considering the pre-pandemic and the pandemic period (social distancing measures). To assess physical activity, caregivers were asked to estimate the youth's mean daily time (in hours) of physical activity practiced before the pandemic and during quarantine. Physical activity was defined as the performance of activities with high energy consumption, such as running, playing soccer, outdoor games, swimming, dancing, etc. Accordingly, the mean daily time (in hours) of screen before and during quarantine was assessed and defined as time spent using electronic devices, namely: television, video game, tablet, smartphone, and computer. Finally, participants were asked to rate (from 0 to 10) the quality of the youth's eating habits and medication adherence before and during quarantine. The assessment of medication adherence was performed only in the diabetes group.

### Outcomes

The primary outcome was the comparison of changes in physical activity time, screen time, and food quality scores from the start of social distancing measures between the diabetes group and the control group.

### Sample Size

The strategy proposed by Krejcie and Morgan was used to calculate the sample size, allowing sample calculations to be conducted for web-based research based on the prevalence of the disease in the population (15). The known prevalence of 95,800 individuals with diabetes younger than 18 years in Brazil in 2019 was used (16). For an analysis using a 95% confidence level and a 0.05 margin of error, 380 responses in the diabetes group would be needed for the adequate power for the proposed analyses. The strategy was repeated for the control group.

### Statistical methods

Data were transcribed from the online platform SurveyMonkey (San Mateo, CA, USA; http://www.surveymonkey.com) to the Statistical Package for Social Science (SPSS®) version 20 for analysis. Participants’ sociodemographic characteristics data were reported as mean ± standard deviation (SD), if the assumption of normal distribution did not seem violated, and prevalence n (%). Differences between groups were assessed by the unpaired t-test for baseline data and Chi-Square tests for categorical variables.

The multiple imputation algorithm was used for the missing data. Data of caregivers’ perceptions of changes in habits during the pandemic were reported as median ± interquartile range (IQR). Analyses were performed using the Mann-Whitney U test for between groups comparisons and the Wilcoxon Rank test for within-group comparisons. A post hoc analysis was performed using logistic regression to correct for confounding factors identified in the baseline characteristics (ethnicity, income, region of origin, and age). The results are presented as odds ratio (OR) and their respective 95% confidence interval (95%CI). Furthermore, an exploratory analysis was performed according to age groups: <12 years (children) and ≥12 years (adolescents). According to the Article 2 of the Brazilian Statute of Children and Adolescents (ECA), those aged up to 12 years are considered children and those between 12 and 18 years old are considered adolescents (17). This was also analyzed by logistic regression and with correction for the different baseline factors between the diabetes and control groups (ethnicity, income, region of origin, and age). Statistical significance was considered as P ≤ 0.05.

## RESULTS

### Participants’ characteristics

In total, 1,011 responses to the online questionnaire were received (485 from caregivers of children and adolescents with diabetes and 526 from caregivers of children and adolescents without diabetes). Enrollment finished after more than 380 participants in each group met the inclusion criteria and answered more than 75% of the proposed questionnaire, finalizing the study sample. Representatives from all regions of Brazil were included, with many participants from the South/Southeast in both groups. The mean age of participants was 39.9 ± 8.6 years; 95.2% were women and 47.8% had low-medium family income. In both groups, most participants were mothers (89.1%). The diabetes and control groups differed regarding ethnicity (white people: 68.8% vs. 87.7% in the diabetes and the control group, respectively; P < 0.001) and family income (low-middle income: 54.1% vs. 41.5% in the diabetes and control group, respectively; P = 0.001) (Table 1).

**Table 1:** Demographics and clinical characteristics of study participants

	Total (n = 764)			Diabetes group (n = 381)	Control group (n = 383)	P-value
Age (years)	39.9 ± 8.6			40.3 ± 8.1	39.5 ± 9.0	0.19
Sex (% female)	727 (95.2)			363 (95.3)	364 (95.0)	0.88
Race/ethnicity (% white)	598 (78.3)			262 (68.8)	336 (87.7)	<0.001
Lower-middle income*	365 (47.8)			206 (54.1)	159 (41.5)	0.001
Parentage (% mother)	681 (89.1)			342 (89.8)	339 (88.5)	0.17
Main caregiver	611 (80.4)			308 (81.7)	303 (79.1)	0.37
Age of the youth (years)	10.0 ± 4.9			11.9 ± 4.3	8.2 ± 4.7	<0.001
Age range of the youth <12 years	445 (58.2)			165 (43.3)	280 (73.1)	<0.001
**Youth with type 1 diabetes characteristic**						
Disease duration (years)				5.0 ± 3.8		
HbA1c (%) mmol/mol				8.1 ± 1.4 65.0 ± 15.3		
Basal bolus insulin use				301 (79.0)		
Capillary blood glucose monitoring				270 (70.9)		
Hypoglycemia (>3 times a week)				109 (28.6)		
Chronic complications				34 (8.9)		
**Psychosocial assessment and routine changes during the pandemic**						
Follows social distancing		725 (94.9)		366 (96.1)	359 (93.7)	0.14
Suspended in-class activities		716 (93.7)		353 (92.7)	363 (94.8)	0.22
Suspended outside activities		634 (83.0)		328 (86.1)	306 (79.9)	0.02
Difficulty in medical assistance		237 (31.0)		164 (43.0)	73 (19.1)	<0.01

Data are mean ± standard deviation or n (%). α ≤ 0.05 indicates significant difference.

* Lower-middle income: family that earns less than 2,564 reais per month as defined by the Strategic Affairs Secretariat (SAE) of Brazil in 2012, equivalent to 495.8 dollars or 430 euros.

For the dependent children or adolescents, the mean age was different between groups: 11.9 ± 4.3 years in the diabetes group and 8.2 ± 4.7 years in the control group (P < 0.001). Regarding diabetes characteristics, the mean disease duration was 5.0 ± 3.8 years and the HbA1c was 8.1% ± 1.4% (65 ± 15.3 mmol/mol). In total, 79.0% of the youth were undergoing basal-bolus insulin regimen and 70.9% monitored capillary blood glucose daily. Moreover, 28.6% reported episodes of hypoglycemia more than three times a week and 8.9% stated having chronic complications (Table 1).

Regarding routine changes during the pandemic, 94.9% of participants followed the social distancing guidance. As for activities, most participants reported the suspension of in-classroom school activities (92.7% of the diabetes group and 94.8% of the control group). The groups differed regarding the suspension of outside activities (reported by 86.1% of the diabetes group and 79.9% of the control group, P = 0.02). As expected, the diabetes group had greater difficulty in medical assistance than the control group (43.0% vs. 19.1%, P < 0.01) (Table 1).

### Physical activity time

Caregivers were asked to estimate the time, in hours, of physical activity performed by the children and adolescents before and during the period of social distancing. The diabetes group already had a reduced median time of physical activity before the pandemic compared to the control group [median 2.0h (IQR 1.0-3.8) diabetes group vs. 3.0h (2.0-5.0) control group, P < 0.001], which continued during the pandemic [1.0h (0.0-1.0) diabetes group vs. 1.0h (0.0-2.0) control group, P < 0.001] (Table 2).

**Table 2:** Perceptions about changes in behavioral and dietary parameters identified by caregivers before and after the COVID-19 pandemic began

		Diabetes group (n = 381)	Control group (n = 383)	P-value
Physical activity time† (hours)				
	Before	2.0 (1.0-3.8)	3.0 (2.0-5.0)	<0.001
	After	1.0 (0.0-1.0)	1.0 (0.0-2.0)	<0.001
Change between periods		-1.0 (-2.0-0.0)	-1.0 (-3.0-0.0)	0.58
P-value (within-group)		<0.001	<0.001	
Screen time† (hours)				
	Before	3.0 (2.0-5.0)	2.0 (2.0-4.0)	<0.001
	After	6.0 (4.0-9.0)	5.0 (3.0-8.0)	<0.001
Change between periods		3.0 (1.0-5.0)	2.0 (1.0-4.0)	0.03
P-value (within-group)		<0.001	<0.001	
Quality of eating habits¥ (score)				
	Before	8.0 (7.0-9.0)	8.0 (7.0-9.0)	0.31
	After	7.0 (5.1-8.1)	8.0 (6.0-9.0)	<0.001
Change between periods		0.0 (-2.0-0.0)	0.0 (-1.0-0.0)	<0.01
P-value (within-group)		<0.001	<0.01	
Treatment adherence¥ (score)				
	Before	6.8 (1.0-9.0)		
	After	7.8 (1.0-8.0)		
Change between periods		0.0 (-0.5-0.0)		
P-value (within-group)		P = 0.60		

Data are median and interquartile range (IQR) of the number of hours or scores reported by caregivers about the two evaluated periods.

† Participants were asked to estimate how many hours, on average, children and adolescents performed these activities before and during the COVID-19 pandemic.

¥ The participants were asked to score, from 0 to 10, the quality of the youth's daily routine before and during the COVID-19 pandemic.

α ≤ 0.05 indicates significant difference.

Regarding within-group comparisons, the median time of physical activity decreased in both diabetes and control groups [diabetes group: median change between periods of −1.0h (IQR −2.0-0.0), P < 0.001, vs. control group: −1.0h (−3.0-0.0), P < 0.001]. The groups had equal median change in the time of physical activity considering the pre-pandemic and pandemic periods (P = 0.58 for between group analysis) (Table 2).

An exploratory analysis was conducted to assess the changes in the time of physical activity, stratifying the groups between children and adolescents. After correcting for ethnicity, family income, child's age, and region of origin, compared to the control group, the children in the diabetes group had an adjusted OR 1.19 (95%CI: 0.73-1.95) for the probability of reduced physical activity time and adolescents had an OR 0.78 (0.42-1.42) (Table 3).

**Table 3:** Perceptions about worse behavioral and dietary parameters of children and adolescents, identified by caregivers

	Diabetes group	Control group
**Child (< 12 years old)**	**n = 165**	**n = 280**
Reduced physical activity time, n (%)	123 (74.5)	189 (67.5)
OR (95%CI)	1.19 (0.73-1.95)	1
Increased screen time, n (%)	133 (80.6)	218 (77.9)
OR (95%CI)	0.81 (0.47-1.39)	1
Worse eating habits, n (%)	61 (37.0)	75 (26.8)
OR (95%CI)	1.58 (1.01-2.49)	1
**Adolescent (≥12 years old)**	n = 216	n = 103
Reduced physical activity time, n (%)	159 (73.6)	81 (78.6)
OR (95%CI)	0.78 (0.42-1.42)	1
Increased screen time, n (%)	183 (84.7)	90 (87.4)
OR (95%CI)	0.88 (0.42-1.85)	1
Worse eating habits, n (%)	93 (43.1)	34 (33.0)
OR (95%CI)	1.42 (0.84-2.40)	1

Data are frequency n (%) and odds ratio (OR) with confidence interval (95%). All odds ratios are adjusted for the child's age, race/ethnicity, income, and location.

### Screen time

Caregivers were asked to estimate, in hours, children and adolescents’ screen time before and during the period of social distancing. Compared to the control group, the diabetes group already presented an increased median time of screen use before the pandemic [3.0h (2.0-5.0) diabetes group vs. 2.0h (2.0-4.0) control group, P < 0.001], which continued during the pandemic [6.0h (4.0-9.0) diabetes group vs. 5.0h (3.0-8.0) control group, P < 0.001].

Regarding within-group comparisons, the median of screen time increased in both diabetes and control groups [diabetes group: median change of 3.0h (1.0-5.0), P < 0.001 vs. control group: 2.0h (1.0-4.0), P < 0.001]. The diabetes group had worse median change in screen time than the control group between the pre-pandemic and pandemic periods (P = 0.03) (Table 2).

An exploratory analysis was conducted, stratifying the groups in children or adolescents. After correcting for confounders, compared to the control group, children in the diabetes group had an adjusted OR 0.81 (0.47-1.39) for the probability of increased screen time whereas adolescents had an OR 0.88 (0.42-1.85) (Table 3).

### Quality of eating habits

Caregivers were asked to rate, from 0 to 10, the quality of eating habits of the youth before and during the period of social distancing. Both the diabetes and the control group had similar median of quality of eating habits scores [median 8.0 (7.0-9.0) diabetes group vs. 8.0 (7.0-9.0) control group, P = 0.31], which decreased in both groups during the pandemic [7.0 (5.1-8.1) diabetes group vs. 8.0 (6.0-9.0) control group, P < 0.001]. However, the reduction of family income during the pandemic was not associated with worse eating habits (OR 0.8; 95%CI: 0.5-1.1). Supplementary Table 2 shows characteristics associated with worsening of eating habits.

Regarding within-group comparisons, the quality of eating habits scores mainly worsened in the diabetes group [diabetes group: median change of 0.0 (IQR −2.0-0.0), P < 0.001 vs. control group: 0.0 (−1.0-0.0), P < 0.001]. The diabetes group had significantly worse median change in eating habits than the control group between the pre-pandemic and pandemic periods (P < 0.01 for between group analysis) (Table 2).

An exploratory analysis was performed, stratifying the groups in children or adolescents and correcting for confounders. Compared to the control group, children in the diabetes group had an adjusted OR 1.58 (1.01-2.49) for the probability of worse eating habits whereas adolescents had an OR 1.42 (0.84-2.40) (Table 3).

### Treatment adherence in the diabetes group

Only caregivers of children and adolescents with diabetes were asked to rate, from 0 to 10, their perceptions on the youth's quality of treatment adherence before and during social distancing. The group presented a median treatment adherence score of 6.8 (1.0-9.0) before the pandemic and a score of 7.8 (1.0-8.0) during the pandemic. The within-group change in the median treatment adherence score remained equal between the pre-pandemic and pandemic periods (P = 0.60 for within-group analysis) (Table 2).

## DISCUSSION

The COVID-19 pandemic changed the daily routine of children and adolescents, including those with type 1 diabetes. This study sought to identify caregivers’ perceptions about the changes in habits of children and adolescents with type 1 diabetes during the social distancing period. Overall, caregivers of youth with and without diabetes perceived a reduced practice of physical activity and increased screen time, reflecting a tendency toward more sedentary habits during the pandemic. Both groups were equally affected regarding physical activity time, but the diabetes group was slightly more affected regarding screen time. Although the eating habits of both groups were affected, caregivers of youth with diabetes indicated that this group's diet was significantly compared to those without diabetes. According to the results of the exploratory analysis, this worsening seems to be mediated mainly by the habits of children aged under 12 years.

Overall, the eating habits of children and adolescents both with and without diabetes worsened during the COVID-19 pandemic. However, parents of youth with diabetes perceived greater impact in comparison to parents in the control group. A study by Cuschieri and Grech indicated that the stress from the schools closure and social distancing measures result in a greater intake of caloric foods (18). Other studies suggest that boredom and anxiety caused by social isolation could induce the youth adopt irregular eating patterns and frequent snacking (14), habits associated with poor metabolic outcomes in adolescents (19). A recent study also conducted in Brazil assessed the eating habits of youth without diabetes during social isolation. The results show that one-third of the participants, especially adolescents, replaced large meals with snacks, mainly those with high fat and sugar content when associated with television-watching. Interestingly, the study also showed that families who respected social distancing (measure associated with higher purchasing power in the study) consumed more raw salad and vegetables, associated with children's lower exposure to unhealthy food environments, such as school cafeterias. Finally, similarly to our results, the study participants presented an excessive screen time and a high prevalence of physical inactivity (20).

Youth with diabetes will likely feel stressed and worried more often considering the challenges of diabetes care during a pandemic and the concern about a possible association between diabetes and the worse outcomes of COVID-19. These emotions can affect eating habits, partially justifying the worsening perceived by caregivers. Moreover, young people with type 1 diabetes are more predisposed to eating disorders, which could also explain why situations of emotional burden worsen eating habits (21). Moreover, since their caregivers are also concerned with possible chronic diseases, these youth could end up overprotected, greatly suffering the consequences of isolation and loneliness.

In this study, caregivers reported a significant increase in screen time in both diabetes and control groups, corroborating studies which assessed children and adolescents without diabetes during the COVID-19 pandemic. Schmidt and cols. found that the screen time of children aged four to 13 years increased up to 60.7 minutes a day during the COVID-19 pandemic (22). This data is especially significant considering that increased screen time is associated with obesity and reduced time of physical activity, directly affecting the glycemic control of diabetes (19,23,24). Similarly, a study by Hourani and cols. conducted with children and adolescents in Jordan found that the COVID-19 lockdown affected these groups’ lifestyle, with an increased food consumption and screen time and reduced their physical activity (25). The authors also found that the mean body weight and body mass index for age Z-scores in children and adolescents significantly increased before and after lockdown (25). The increased screen time seems to result from two factors: the first is home isolation, which requires using electronic devices for distraction and playful activities to escape from boredom; the second is the transition from the school methodology to online classes and activities, which demands using computers and laptops, resulting in more screen time.

Our results show that caregivers from the diabetes group noticed a slightly worse change in screen time than caregivers from the control group. Considering the concerns related to the risk of COVID-19 in patients with chronic diseases, extra care may have resulted in greater isolation, in which electronic devices were often used as entertainment. Other authors have also reported on adolescents’ increased time of electronic device use (22,26). Despite bringing physical and neuro-psychological consequences to health, screen time can help search for a better quality of life for adolescents (27). A study by Nagata and cols. revealed that using social media for up to two hours a day is associated with higher levels of social connectivity in high school students, possibly being a useful tool during the pandemic (28).

The COVID-19 pandemic affected the practice of physical exercise in youth with type 1 diabetes and without diabetes and different age groups. A study by Cancello and cols. showed that only 14% of individuals who practiced more than two hours of physical activity per week maintained their usual routine (29). Similarly, Dunton and cols. found that youth without diabetes decreased their time of physical activity due to schools closure, cancellation of collective practices, and impossibility of interpersonal contact (30). Furthermore, a study by Xiang and cols. found that the physical activity time of children and adolescents used to be around nine hours per week but decreased to less than two hours when social distancing began, showing how confinement significantly affects the youth (31). Effects of the pandemic on the physical activities of children and adolescents are evident, reflecting the need to adapt exercises to at-home practices to maintain a similar routine to that before the pandemic.

The change in habits of youth with diabetes during the pandemic, mainly regarding poor quality of eating habits, make us reflect on possible strategies for encouraging a healthier diet in future situations. Parents are greatly responsible for ensuring that children maintain healthier eating habits. Those responsible for children with diabetes should try offering more healthy options at mealtimes and avoid buying industrialized food. They should also offer children playful activities that encourage eating healthy food, such as inviting them to help prepare their own meal, to motivate them to eat better. On the other hand, adolescents with diabetes are mainly responsible for ensuring their own healthy eating. However, the school and caregivers must provide objective and reliable information on the importance of healthy eating habits for youth with diabetes so these adolescents can realize how much their health depends on a healthy lifestyle, thus changing their diet.

This study has limitations. Considering the absence of validated questionnaires for the Brazilian population, an adaptation of internationally validated questionnaires was used. However, no validation study was conducted before applying the questionnaires to the proposed population while considering the current circumstances of the pandemic, which could have affected the assessment of certain parameters. As an alternative, a pilot study could be conducted with the applied questionnaire, but this could delay the release of results and harm the population that would benefit from them. Moreover, this study is cross-sectional, so it could not establish a cause-and-effect relationship between the found associations. Ideally, pre- and post-pandemic studies should be conducted longitudinally to make inferences with greater certainty. However, since the pandemic was unexpected, previous assessments could not be conducted at the proper timing. Furthermore, we did not objectively document the assessment of physical activity and screen time or scores for food quality and treatment adherence, which were based on the perceptions of caregivers. This could be a possible source of bias since caregivers of youth with diabetes tend to focus more on their children's habits, reporting some aspects more negatively. This study also did not assess the loss of loved ones, which could have interfered with the results. Additionally, most participants are female caregivers from South and Southeast Brazil, a limitation for the external validity of the results found. Analyses were conducted to correct different baseline characteristics between groups, including differences in ethnicity, income, region of origin, and age of the dependent, reducing their interference. Finally, we emphasize that since this is a cross-sectional study conducted with an online survey, most measurements were subjective, hindering the assessment of self-reported laboratory and weight outcomes such as body mass index (BMI), weight gain, and glycemic control. We therefore could not associate worsening of habits with worsening of glycemic control and weight gain nor assess if the participants’ BMI influenced the results.

Maintaining healthy habits daily is essential to manage type 1 diabetes. The pandemic period changed the daily routine, which could be significantly harmful to children and adolescents with diabetes. The creation of virtual support groups is a possible alternative to mutually encourage young people and caregivers to seek a healthy way of life during the pandemic. Ideas of indoor physical activities that can be performed with the youth could also stimulate these groups. We must constantly adapt to the context in which we currently live and be willing to develop new healthy habits. The COVID-19 pandemic is just another challenge for those who have a chronic disease, and strategies to improve life habits are essential to remain healthy during and after the outbreak.

## Supplementary Table 1 Questionnaire applied in the methods

**Table:**

1	What is your gender?
2	What is your gender?
3	What's your color?
4	What is your marital status?
5	What is your family's average monthly income?
6	In which state do you live?
7	In which city do you live?
8	What is your birth year?
9	What is the bond you have with the child/adolescent in question?
10	What is the year of birth of the child or adolescent you are responsible for?
11	Does the child/adolescent have any chronic health problems?
12	If you answered yes to the previous question, how old was the child when he received this diagnosis?
13	Does the child/adolescent have any cognitive limitations or do they need continuous health care for basic activities?
14	Does the child/adolescent use medication continuously? If yes, which ones?
15	Did the child get the flu vaccine?
16	Are you primarily responsible for the care of the child/adolescent in question?
17	Do you have people you can count on to help with the child/adolescent's care (partner, grandparents, friends…)?
18	Do you maintain satisfying social relationships, such as regular contact with friends and trusted people? (Take into account the relationships you maintained in the period prior to the current COVID-19 pandemic to answer this question)
19	Do you find your home environment welcoming (ie, do you feel that you can freely express your feelings to people in your family, feel understood, and receive support when needed)?
20	Are you following the social distancing orientation?
21	Have the in-school activities of the child or adolescent for whom you are responsible been suspended?
22	Does the child or teenager for whom you are responsible maintain any activity outside the home during the period of social distancing?
23	What is the average time – in HOURS – of PHYSICAL ACTIVITY performed DAILY by the child/adolescent (activities involving high energy consumption such as running, playing ball, outdoor games, swimming, dancing, etc.)?
24	What is the average time – in HOURS – that the child/adolescent spends using ELECTRONIC DEVICES DAILY? (time spent with television, video game, tablet, smartphone and computer).
25	What grade – from 0 to 10 – would you give to the FOOD HABITS of the child/adolescent (considering aspects such as food quality, nutritious meals and adequate amounts)?
26	If the child/adolescent uses any medication continuously, what grade – from 0 to 10 – would you give for medication adherence (considering the correct use of medications in relation to the prescribed doses and the indicated times)? If your child is not taking medications, answer ZERO.
27	Was anyone in your family a suspected or confirmed case of COVID-19?
28	Was the child/adolescent in your care a suspected or confirmed case of COVID-19?
29	Did your family's income change during the COVID-19 pandemic?
30	Have you had difficulties in getting supplies for the child or adolescent for whom you are responsible (buying varied foods, medications, etc.)?
31	Have you had difficulties in getting medical care for the child or adolescent for whom you are responsible during the COVID-19 pandemic?

## Supplementary Table 2 Characteristics associated with worsening of eating habits

**Table:**

	Odds ratio	95% Confidence Interval	P Value
Suspected case of COVID-19 in the family	0.8	0.5-1.3	0.29
Income reduction	0.8	0.5-1.1	0.15
Difficulty in medical assistance	0.7	0.5-1.0	0.06
Increased screen time	2.1	1.4-3.3	<0.001
Follows social distancing	0.9	0.4-1.8	0.78
Suspension of school activities	1.5	0.7-3.0	0.26
Type 1 diabetes	1.6	1.1-2.2	<0.01

Data are OR and 95% confidence interval.
